# Immunity of the *Saccharomyces cerevisiae SSY5* mRNA to nonsense-mediated mRNA decay

**DOI:** 10.3389/fmolb.2014.00025

**Published:** 2014-12-08

**Authors:** Jesseeca Obenoskey, Dakota R. Lane, Audrey L. Atkin, Bessie W. Kebaara

**Affiliations:** ^1^Department of Biology, Baylor UniversityWaco, TX, USA; ^2^School of Biological Sciences, University of Nebraska-LincolnLincoln, NE, USA

**Keywords:** 3′-untranslated regions, nonsense-mediated mRNA decay, *SSY5*, mRNA decay, mRNA stability

## Abstract

The nonsense-mediated mRNA decay (NMD) pathway is a specialized pathway that triggers the rapid degradation of select mRNAs. Initially, identified as a pathway that degrades mRNAs with premature termination codons, NMD is now recognized as a pathway that also regulates some natural mRNAs. Since natural mRNAs do not typically contain premature termination codons, these mRNAs contain features that target them to NMD. In *Saccharomyces cerevisiae* mRNAs with atypically long 3′-UTRs are usually degraded by NMD, however in some conditions a constitutively expressed *SSY5* mRNA with multiple NMD targeting signals including an atypically long 3′-UTR is an exception. We investigated the features of the *SSY5* mRNAs that confer immunity to NMD. We found that the *SSY5* mRNA 3′-UTRs are sufficient to target NMD insensitive mRNA to the pathway. Replacing the *SSY5* 3′-UTRs with the *cyc1-512* 3′-UTRs, known to target mRNAs to NMD or with the *CYC1* 3′-UTR, known not to target mRNAs to NMD, resulted in production of *SSY5* mRNAs that were regulated by NMD. These observations suggest that the *SSY5* mRNAs require sequences both within the 5′-UTR and/or ORF as well as the 3′-UTR to escape decay by NMD.

## Introduction

The nonsense-mediated mRNA decay (NMD) pathway is conserved in all eukaryotic organisms that have been examined so far. It elicits the rapid degradation of mRNAs with premature termination codons and, importantly, some natural mRNAs as well (Reviewed in Culbertson and Leeds, 2003; Amrani et al., 2006). Three core trans-acting factors are required for NMD in all eukaryotes. These are the up-frameshift proteins Upf1p, Upf2p, and Upf3p. The essential role of these proteins in this pathway was initially discovered in the yeast *Saccharomyces cerevisiae* and later found in multicellular eukaryotes. Elimination of any one of these three proteins selectively stabilizes mRNAs that are targeted for NMD-mediated degradation.

The regulation of natural mRNAs by NMD has been observed in multiple organisms ranging from yeast to humans. In global analysis studies of the effect of NMD on transcript levels in *S. cerevisiae* (Lelivelt and Culbertson, 1999; He et al., 2003; Guan et al., 2006; Johansson et al., 2007), *D. melanogaster* (Rehwinkel et al., 2005) and humans (Mendell et al., 2004; Whittmann et al., 2006; Yepiskoposyan et al., 2011) ~10% of the transcriptome is affected when NMD is non-functional. The majority of the affected mRNAs accumulate in *nmd* mutants (Lelivelt and Culbertson, 1999; He et al., 2003; Guan et al., 2006; Johansson et al., 2007).

There are several signals that are known to activate the decay of natural mRNAs by NMD. They include: (1) Translated upstream open reading frames (uORFs) (Gaba et al., 2005; Guan et al., 2006). (2) Translation initiation at an out-of-frame AUG within the main open reading frame, also referred to as leaky scanning (Welch and Jacobson, 1999; Guan et al., 2006), (3) Inefficiently spliced pre-mRNAs (He et al., 1993; Guan et al., 2006). (4) Some non-productive alternatively spliced transcripts (Kawashima et al., 2014). (5) ribosomal frameshifts (Belew et al., 2010). (6) an Upf1p-dependent destabilizing element (UDE) has been shown to cause degradation of the *PPR1* mRNA by NMD (Kebaara et al., 2003b), and (7) atypically long 3′-UTRs (Muhlrad and Parker, 1999; Singh et al., 2008; Kebaara and Atkin, 2009; Yepiskoposyan et al., 2011). Collectively, these studies show that there are a variety of known signals that induce degradation of mRNAs by NMD. Most of these NMD-targeting features are conserved in other organisms.

Currently three natural *S. cerevisiae* mRNAs containing NMD targeting signals, are known to be immune to the pathway (Ruiz-Echevarria and Peltz, 2000; Kebaara and Atkin, 2009). These NMD-resistant mRNAs include *SSY5* mRNA, a natural mRNA with an atypically long 3′-UTR (Kebaara and Atkin, 2009), as well as *GCN4* and *YAP1* mRNAs which have uORFs (Ruiz-Echevarria and Peltz, 2000). That some endogenous mRNAs with NMD targeting features can escape NMD suggests that these mRNAs have evolved mechanisms to evade degradation by the pathway.

We originally identified *SSY5* mRNA as a potential NMD substrate in a screen for *S. cerevisiae* mRNA with atypically long 3′-UTRs. *SSY5* mRNAs have 3′-UTRs of ~475 nucleotides. This is atypically long for *S. cerevisiae* 3′-UTRs. In *S. cerevisiae* 3′-UTRs typically range in size from 50 to 200 nt, with a median length of 121 nt. The atypically long *SSY5* 3′-UTRs would be expected to target the mRNAs to the NMD pathway. However, in the conditions examined initially, we found that *SSY5* mRNAs are not degraded by the pathway (Kebaara and Atkin, 2009).

Here, we investigated the immunity of the *SSY5* mRNAs to the NMD pathway. We show that the *SSY5* mRNA is immune to NMD in some conditions. Upon exposure to heat stress this *SSY5* mRNA becomes sensitive to the NMD pathway in some genetic backgrounds, while a second, shorter *SSY5* mRNA is insensitive to the pathway. However, the long *SSY5* mRNA 3′-UTR is sufficient to target an NMD-insensitive mRNA for NMD induced degradation in all conditions tested. Further, we demonstrate that replacing the *SSY5* 3′-UTR with the *cyc1-512* 3′-UTR, which has been shown to target NMD-insensitive mRNAs to the pathway, results in production of fusion mRNAs that were regulated in an NMD-dependent manner. Additionally, replacement of the *SSY5* 3′-UTR with the *CYC1* 3′-UTR, which does not target mRNAs to the NMD pathway, also resulted in mRNAs that were regulated by NMD. These observations suggest that the *SSY5* mRNA requires sequences within both the 5′-UTR and/or ORF as well as the 3′-UTR to escape decay by the NMD pathway.

## Materials and methods

### Yeast strains

The *S. cerevisiae* strains used in this study were, W303a (MATa ade2-1 ura3-1 his3-11,15 trp1-1 leu2-3,112 can1-100), AAY320 (MATa ade2-1 ura3-1 his3-11,15 trp1-1 leu2-3,112 can1-100 upf1-Δ2), AAY334 (MATa ura3-1 his3-11,15 trp1-1 leu2-3,112 rpb1-1), AAY335 (MATa ura3-1 his3-11,15 trp1-1 leu2-3,112 rpb1-1 upf1-Δ2) and B4060 (MATa lys2 his1 trp1 cyc1-512) (Wente et al., 1992; Kebaara et al., 2003a). Yeast strains were grown and maintained using standard methods (Ausubel et al., 1998). Plasmids were maintained in E. coli DH5α and transformed into yeast strains using Lithium Acetate mediated transformation (Gietz and Woods, 1998).

### Heat shock treatment

*S. cerevisiae* strains W303a (*UPF1*), AAY320 (*upf1*Δ), AAY334 (*UPF1 rpb1-1*) and AAY335 (*upf1Δ rpb1-1*) were grown at 28°C in rich medium (YPD) to mid-log phase according to our mRNA steady-state accumulation protocol (Kebaara et al., 2012). The yeast strains were then transferred to a 39°C water bath for a 1 min heat shock before harvesting. Subsequently, RNA was extracted from the yeast strains as described below in the RNA methods.

### DNA methods

The DNA constructs used in this study were generated using cloning free PCR (Erdeniz et al., 1997). The primers used to generate the PCR fragments are listed in Table 1. Yeast genomic DNA from W303 or B4060 was used as the template to generate the PCR fragments. For each construct two separate PCR fragments were generated. One fragment encompassed the 5′-UTR and ORF and the second fragment contained the 3′-UTR. The 5′-UTR and ORF fragments were then fused to the 3′-UTR fragment using cloning free PCR. The 5′-UTR and ORF fragments generated by PCR were for *CYC1* and *SSY5* genes using the primers listed in Table 1. The *CYC1* 5′-UTR and ORF fragment was fused to the *SSY5* 3′-UTR to generate the *CYC1-SSY5* 3′-UTR fragment. The *SSY5* 5′-UTR and ORF was fused to either the *CYC1* 3′-UTR or the *cyc1-512* 3′-UTR to generate the *SSY5-CYC1* 3′-UTR fragment and the *SSY5-cyc1-512* 3′-UTR fragment respectively. The fusion DNA fragments were then inserted into the TOPO-TA cloning vector according to the manufacturer's instructions (Life Technologies, Grand Island, NY). All DNA fusion constructs generated by PCR were sequenced to confirm that they were error free and that precise fusions were generated. After sequencing the fusion constructs were subcloned out of the TOPO-TA cloning vector and inserted into the high copy yeast vector pRS425 (Sirkosky and Heiter, 1989).

**Table 1:** **Primers used to generate DNA constructs and determine 3′-UTR lengths**.

**Primer**	**Sequence**	**Utilized for**
*CYC1* 5′	5′ TAAATATTCTTTCCTTATACATTAG 3′	CYC1 5′-UTR and ORF
*SSY5CYC1ORF*	5′CATCCTGTAATGGGTTAAATAACTTCAAAAAGGCAAATTACTCACAGGCTTTTTTCAAG 3′	CYC1 5′-UTR and ORF with primer CYC1 5′
*CYC1-SSY5 3*'*-UTR*	5′AAACGACTTAATTACCTACTTGAAAAAAGCCTGTGAGTAATTTGCCTTTTTGAAGTTATTTAAC 3′	SSY5 3′-UTR with primer SSY5 3′-UTR
*SSY5* 3′-UTR	5′ ACATAGTTGTAGAATCAGAAATC 3′	SSY5 3′-UTR with primer CYC1-SSY5 3′-UTR
*SSY5* 5′	5′ CGTTAATTTTACGCTCGAGGTAC 3′	SSY5 5′-UTR and ORF with primer CYC1-SSY5
*CYC1-SSY5*	5′TAACTAATTACATGATATCGACAAAGGAAAAGGGGCCTGTTCATCCATCTAGTTGT-3′	SSY5 5′-UTR and ORF fused to the CYC1 or cyc1-512 3′-UTR with primer SSY5 5′
*SSY5-CYC1 3*'*-UTR*	5′GTAACTAAAATTCAATGGGACATTGATCCACAACTAGATGGATGAACAGGCCCCTTTTCCTTTGTC 3′	CYC1 and cyc1-512 3′-UTR with CYC1 and cyc1-512 3′-UTR primer
*CYC1 and cyc1-512 3*'*-UTR*	5′ CTTGTCGCTTCCATTCGTTG 3′	CYC1 and cyc1-512 3′-UTR
*SSY5 3*'	5′GATCCACAACTAGATGGATGA 3′	SSY5 3′ RACE
*SSY5 3*'nested	5′TTTGCCTTTTTGAAGTTATTTAAC 3′	SSY5 3′ RACE nested
*CYC1 3*'	5′ TGAAAAAAGCCTGTGAGTAA 3′	CYC1 3′ RACE
*CYC1 3*' nested	5′ CTGTGAGTAAACAGGCCCCT 3′	CYC1 3′ RACE nested

### RNA methods

Yeast total RNA used for mRNA steady-state accumulation measurements was extracted by the hot phenol method from yeast strains, W303 and AAY320 harvested at mid-log phase (Kebaara et al., 2003a). Total RNA used to measure mRNA decay rates, was harvested as described in Kebaara et al. (2012). Briefly, yeast strains AAY334 (*UPF1, rpb1-1*) and AAY335 (*upf1Δ, rpb1-1*) were grown at 28°C to mid-log phase. The yeast cells were then transferred to 39°C, the non-permissive temperature. Yeast cells were successively harvested at different time points after transcription was inhibited by the temperature shift and total RNA extracted.

Yeast total RNA was resolved on a 1% agarose-formaldehyde gel followed by transfer of the RNA to Genescreen Plus membranes. The northern blots were probed with oligolabeled DNA probes. DNA templates for probe synthesis were generated by PCR. The probes were labeled with ^32^P using an Oligolabeling kit (Life Technologies, Grand Island, NY). Northern blots were phosphorImaged™ with a Typhoon phosphorImager (Amersham Pharmacia Biotech, Inc.). mRNA decay rates were determined by graphing log_10_ of the percent mRNA remaining vs. time using SigmaPlot 2000, Version 6.10 (SPSS Science, Chicago, IL). The mRNA steady-state levels and decay rates were normalized using *SCR1* RNA, an RNA polymerase III transcript.

### 3′-RACE analysis

3′-RACE analysis was done as described in Kebaara et al. (2012). Briefly, 5 μg of yeast total RNA from W303 or AAY320 used for quantitative northern analysis was utilized to make cDNA using SuperScript™ II RT (Life Technologies, Grand Island, NY). The cDNA was subsequently used as template DNA for the primary PCR reactions using the Abridged Universal Amplification Primer (AUAP) provided with the 3′-RACE kit and a *SSY5* or *CYC1* gene-specific primer. Following the primary PCR reactions, a nested PCR reaction was done using the initial PCR product (primary reaction) as the template and a nested *SSY5* or *CYC1* primer. Both the primary and nested PCR products were run on 1.5% agarose gels for visualization of the 3′-RACE PCR products.

## Results

### The SSY5 mRNA produced in wild-type and NMD mutant yeast strains has a long 3′-UTR, but is not degraded by the NMD pathway while it is regulated by the NMD pathway in identical yeast strains with the rpb1-1 allele

Our previous study demonstrated that most natural mRNAs with atypically long 3′-UTRs are degraded by the NMD pathway (Kebaara and Atkin, 2009). In sharp contrast, we found that *SSY5* mRNAs have atypically long 3′-UTRs but did not accumulate in nmd mutants. *SSY5* 3′-UTRs are predicted to range in size from 384 to 464 nt by the mRNA 3′-processing site predictor (http://harlequin.jax.org/drupal/?q=polyA; Graber et al., 2002). This 3′-UTR length was experimentally confirmed by 3′-RACE and determined to be ~475 nt, using steady-state accumulation total RNA (Kebaara and Atkin, 2009 and Supplementary Figure S1, lane 5). This 3′-UTR is atypically long for *S. cerevisiae* and should target the *SSY5* mRNAs to the NMD pathway.

**Figure 1:**
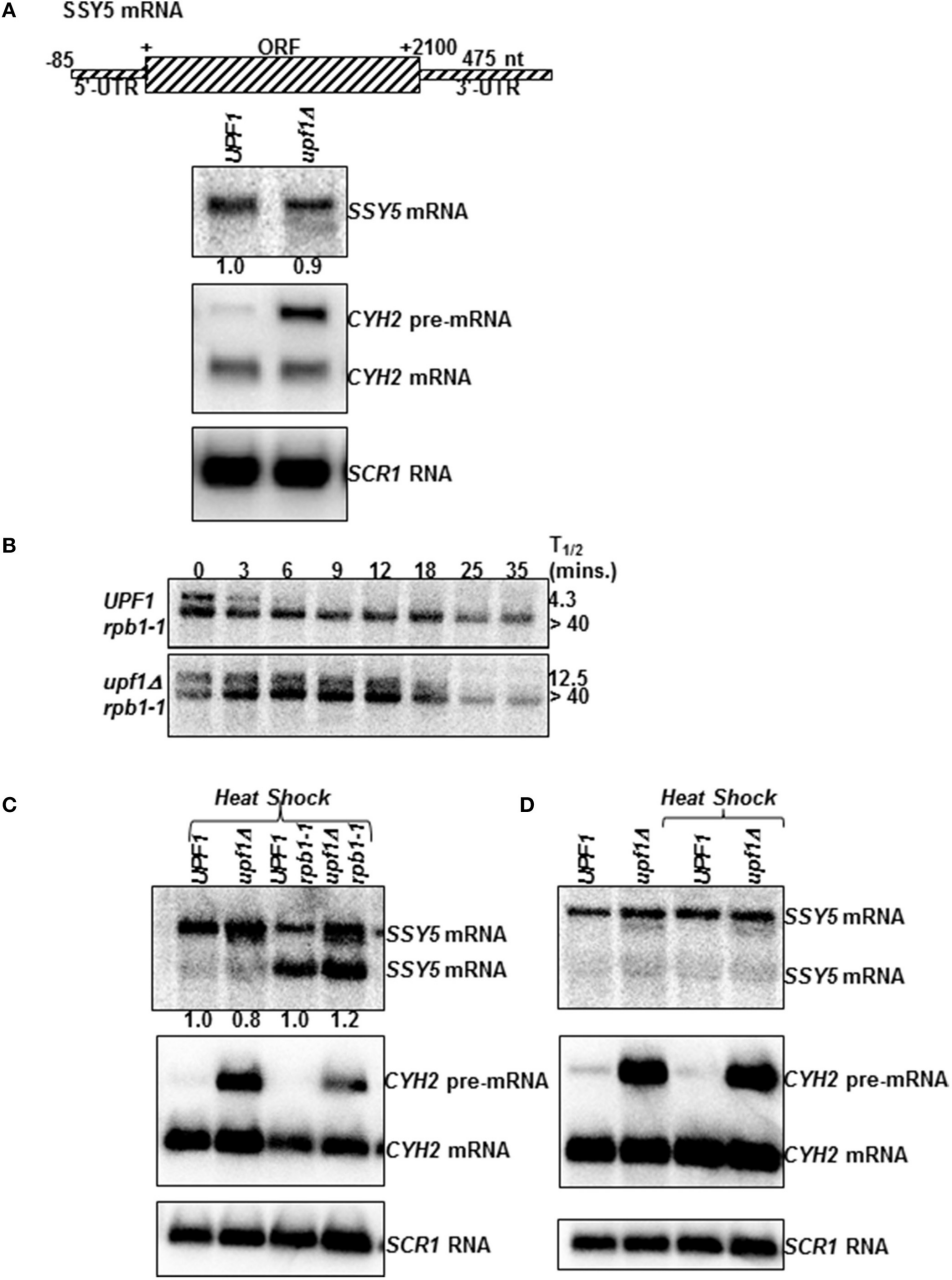
**The *SSY5* mRNA with an atypically long 3′-UTR can evade degradation by the NMD pathway**. mRNA schematic **(A)** and northern blot analysis of the *SSY5* mRNA steady-state levels **(A)**, and half-lives **(B)** in wild-type (*UPF1*) and yeast strains with a non-functional NMD pathway (*upf1Δ*). **(A)** The *SSY5* mRNA steady-state levels were measured from total RNA isolated from W303 (*UPF1*, Wente et al., 1992) and AAY320 (*upf1Δ*, Kebaara et al., 2003a). *CYH2* and *SCR1* were used as controls. *CYH2* pre-mRNA is degraded by NMD and is used to confirm the NMD phenotype of the yeast strains. *SCR1* is a loading control. It is transcribed by RNA polymerase III and is not degraded by the NMD pathway (Kebaara and Atkin, 2009). **(B)** The mRNA half-lives were measured using total RNA isolated from AAY334 (*UPF1 rpb1-1*) and AAY335 (*upf1Δ rpb1-1*) (Kebaara et al., 2003a). mRNA half-life measurements are shown on the right of the representative northern blots. Time after inhibition of transcription is shown above the northern blots. The mRNA half-lives are an average of three independent experiments. **(C)** The *SSY5* mRNA steady-state levels following heat shock were measured from total RNA isolated from W303 (*UPF1*), AAY320 (*upf1Δ*), AAY334 (*UPF1 rpb1-1*) and AAY335 (*upf1Δ rpb1-1*) after exposure to 39°C for 1 min. The steady-state levels of the longer *SSY5* mRNA (top band) are shown below the steady-state northern blot. **(D)** The *SSY5* mRNA steady-state levels from W303 (*UPF1)* and AAY320 (*upf1Δ*) without exposing the yeast strains to heat shock (left two lanes) or after exposure to heat shock (the right two lanes).

*SSY5* mRNA steady-state levels in isogenic wild-type (*UPF1*) and nmd mutant *(upf1Δ)* yeast strains was measured by quantitative northern blotting (Figure 1A). One *SSY5* transcript of ~2.8 kb was detected on steady-state northern blots. *SSY5* mRNA levels were 0.88 ± 0.24 (*n* = 3) in *nmd* mutants *(upf1Δ)* relative to wild-type (*UPF1)* yeast strains. Subsequently, *SSY5* mRNA half-lives were measured in wild-type and *nmd* mutant strains. Unlike the steady-state northern blot, two *SSY5* transcripts were detected on the half-life northerns (Figure 1B). The faster migrating band was slightly shorter than the 2.8 kb *SSY5* transcript. This *SSY5* mRNA had the same half-life in the wild-type *(UPF1)* and *nmd* mutant *(upf1Δ)* strains (Figure 1B). The second *SSY5* transcript detected on the half-life northern was the ~2.8 kb transcript detected on the steady-state northerns and it was now sensitive to the NMD pathway. Notably, we only observed the two *SSY5* transcripts in the conditions and yeast strains used to measure mRNA half-lives. The half-life of the longer *SSY5* transcript was 4.3 ± 1.53 (*n* = 3) min in the wild-type strains and 12.50 ± 0.7 (*n* = 2) min in *nmd* mutant strains (Figure 1B).

*SSY5* mRNA decay rate measurements were done by inhibiting transcription in yeast strains with a temperature sensitive allele of RNA polymerase II (Nonet et al., 1987). The additional *SSY5* transcript could have been produced as a result of the heat shock used to inhibit transcription or due to the yeast strain having a mutation in RNA polymerase II (Nonet et al., 1987). To distinguish between these possibilities, isogenic wild-type and *nmd* mutants with or without the temperature sensitive allele of RNA polymerase II were exposed to a 1 min heat shock at 39°C. 1 min was used because the second *SSY5* band was detected at the zero time point of the half-life measurements (Figure 1B). There was a faint faster migrating band that accumulated to approximately the same levels in wild-type and *nmd* mutant strains (Figure 1C left two lanes). This mRNA was not observed in Figure 1A. In addition the slower migrating *SSY5* mRNA observed in Figure 1A was present. This *SSY5* mRNA was insensitive to the NMD pathway in three independent experiments in this strain background (Figure 1C). As a control, *SSY5* mRNA accumulation levels were compared in wild-type and nmd mutant strain background with or without heat shock (Figure 1D). The ~2.8 kb *SSY5* mRNA was the major transcript observed in these conditions and it was insensitive to NMD. These results show that the shorter, novel *SSY5* mRNA is present primarily in the *rpb1-1* genetic background and is immune to degradation by the NMD.

As discussed above, exposure of the wild-type (*UPF1 rpb1-1*) and *nmd* mutant (*upf1Δ rpb1-1*) with the temperature sensitive allele of RNA polymerase II to heat shock resulted in the production of two transcripts (Figures 1B,C). As observed with the half-life analysis, the longer ~2.8 kb *SSY5* transcript consistently accumulated to slightly higher levels in the *nmd* mutant relative to wild-type strains in the *rpb1-1* background (1.2 ± 0.14 fold, Figure 1C), and the shorter transcript accumulated 0.8 ± 0.4 in the same strain background (Figure 1C). In addition, analysis of the relative accumulation levels of the ~2.8 kb *SSY5* transcript at time zero on the half-life northerns support these results. The ~2.8 kb *SSY5* transcript accumulated 1.4 ± 0.35 fold in *nmd* mutants *(upf1Δ rpb1-1)* relative to wild-type (*UPF1 rpb1-1)* yeast strains, while the shorter transcript accumulated 0.87 ± 0.34 fold in *nmd* mutants (Figure 1B, 0 time point). These results show that the production of an NMD sensitive *SSY5* transcript is associated with the temperature sensitive RNA polymerase II in the *rpb1-1* mutant strains. This is not surprising since RNA polymerase II plays an important role in RNA processing, including 3′-end cleavage and polyadenylation.

These *SSY5* transcripts are differentially sensitive to NMD. The shorter transcript, which has elevated levels in the *rpb1-1* yeast strain after heat shock, is degraded at the same rate in wild-type and *nmd* mutant strains (Figures 1B,C). In contrast, the longer *SSY5* transcript which is the major transcript in wild-type strains is degraded more rapidly in wild-type than *nmd* mutant strains that have the temperature sensitive allele of RNA polymerase II (Figures 1B,C). Thus, this transcript becomes sensitive to NMD in this strain background and under these conditions. The reason why this *SSY5* transcript becomes sensitive to NMD in these conditions is not clear but it could be due to the increased expression of the shorter transcript or due to some indirect effect that alleviates the accumulation of this transcript in nmd mutants. Thus, these results demonstrate that, the *SSY5* mRNA 3′-UTR is insufficient to target all *SSY5* mRNAs for NMD in all conditions tested, while the longer transcript is degraded by the NMD pathway in the *rpb1-1* strain background. The shorter transcript could escape NMD in this strain background because its 3′-UTR may not be sufficiently long to act as an NMD targeting signal even though it is atypically long, or because it is protected from degradation by the NMD pathway.

### The SSY5 mRNA 3′-UTR is sufficient to target an NMD-insensitive mRNA for decay by the pathway in all conditions and yeast strains tested

**Figure 2:**
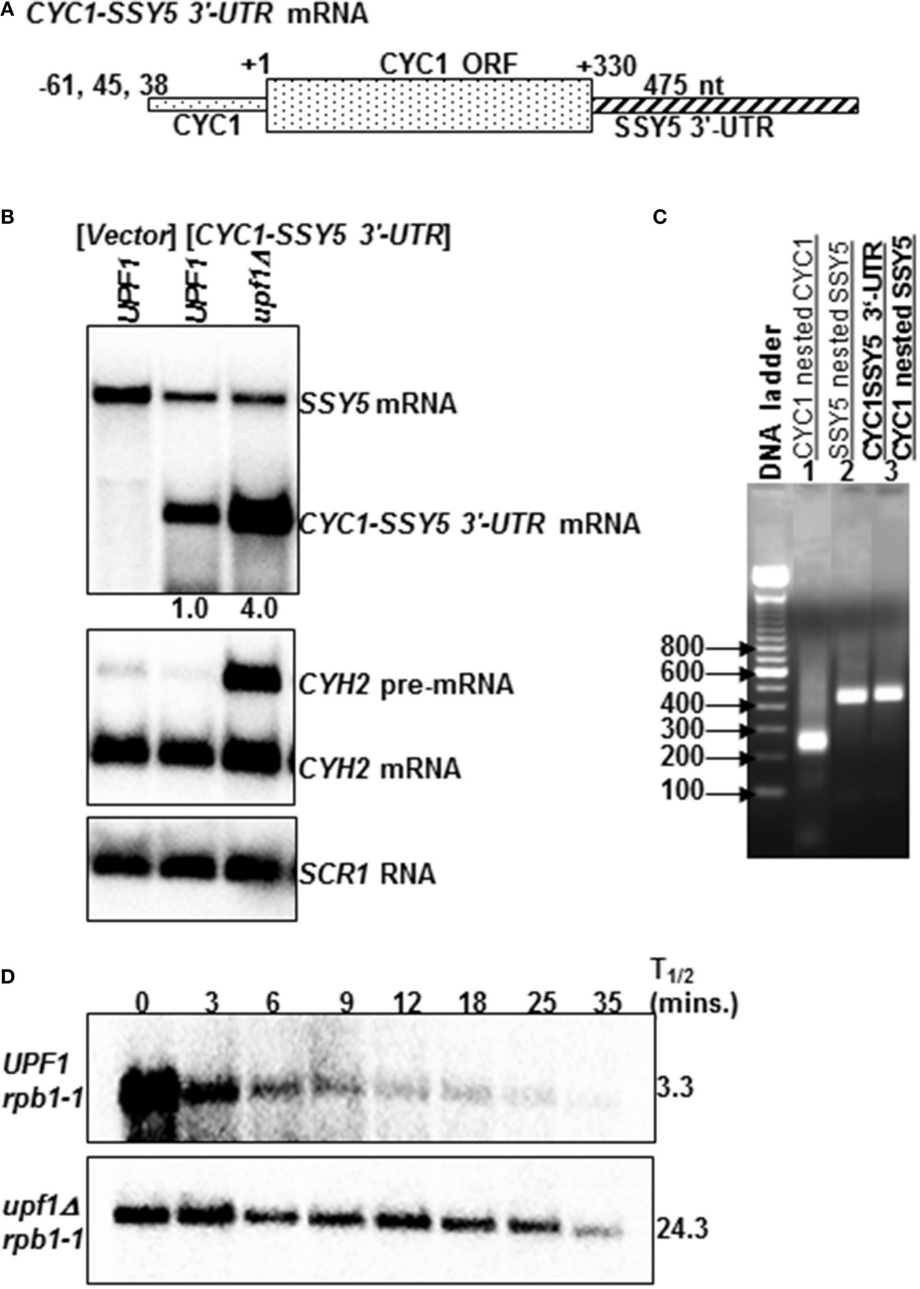
**The atypically long *SSY5* 3′-UTR is sufficient to target an NMD-insensitive mRNA to the NMD pathway**. The schematic diagram represents the *CYC1-SSY5 3*'*-UTR* mRNA **(A)**. This mRNA was used to assess the requirement for the *SSY5* mRNA atypically long 3′-UTR for decay by the NMD pathway **(A)**. *CYC1-SSY5* 3′-UTR mRNA contains the 5′-UTR and ORF of *CYC1*, and the 3′-UTR of *SSY5* **(A)**. Representative steady-state northerns of total RNA isolated from wild-type (*UPF1)* lacking the *CYC1-SSY5 3*'*-UTR* mRNA and wild-type (*UPF1)* and nmd mutants (*upf1Δ)* containing the *CYC1-SSY5* 3′-UTR construct **(B)**. The northern blot was probed with DNA from the *SSY5* 3′-UTR. The relative *CYC1-SSY5* 3′-UTR steady-state levels are shown below the northern blot **(B)**. An agarose gel of 3′ RACE nested PCR products from wild-type yeast strains lacking the *CYC1-SSY5* 3′-UTR mRNA (lanes 1–2) and strains expressing the *CYC1-SSY5* 3′-UTR mRNA (lane 3). Primers used for the 3′ RACE PCR reactions are listed above the lane numbers **(C)**. Half-life measurements of the *CYC1-SSY5* 3′-UTR mRNA are shown in **(D)** and were done as described in Figure 1. mRNA half-life measurements are shown to the right of the northern blots. The steady-state levels and half-life measurements are an average of three independent experiments.

To determine the extent to which the atypically long *SSY5* mRNA 3′-UTR is sufficient to target an NMD insensitive mRNA to the pathway, the 3′-UTR of the *CYC1* mRNA was replaced with the *SSY5* 3′-UTR (Figure 2A). The *CYC1* mRNA, which encodes iso 1 cytochrome C, is normally not degraded by the NMD pathway (Biswadip et al., 2000). The *CYC1* mRNA was selected because it has previously been used to show that abnormally 3′-extended mRNAs resulting from a mutation in the poly(A) site are degraded by the NMD pathway (Muhlrad and Parker, 1999). 3′-RACE and northern blot analysis were utilized to measure 3′-UTR length and determine the steady-state levels of the *CYC1-SSY5 3′-UTR* mRNA. The 3′-UTR of the *CYC1* mRNA was specifically replaced with the *SSY5* 3′-UTR (Figure 2C and Supplementary Figure S1, compare lanes 3 and 8).

The steady-state levels of the *CYC1-SSY5 3′-UTR* mRNA were 4.06 ± 1.42 (*n* = 3) fold higher in *nmd* mutant yeast strains than in wild-type strains (Figure 2B). Additionally, the half-life of the *CYC1-SSY5 3′-UTR* mRNA is longer in the *nmd* mutant strain than the wild-type strain 24.33 ± 4.04 (*n* = 3) min vs. 3.3 ± 1.53 (*n* = 3) min; (Figure 2D). We interpret these results to mean that the *SSY5* mRNA 3′-UTR is sufficient to target the NMD-insensitive *CYC1* mRNA to the NMD pathway and this indicates that the short *SSY5* mRNA is protected from degradation by the NMD pathway.

### The SSY5 mRNAs with abnormally extended cyc1 3′-UTRs are degraded by the NMD pathway

**Figure 3:**
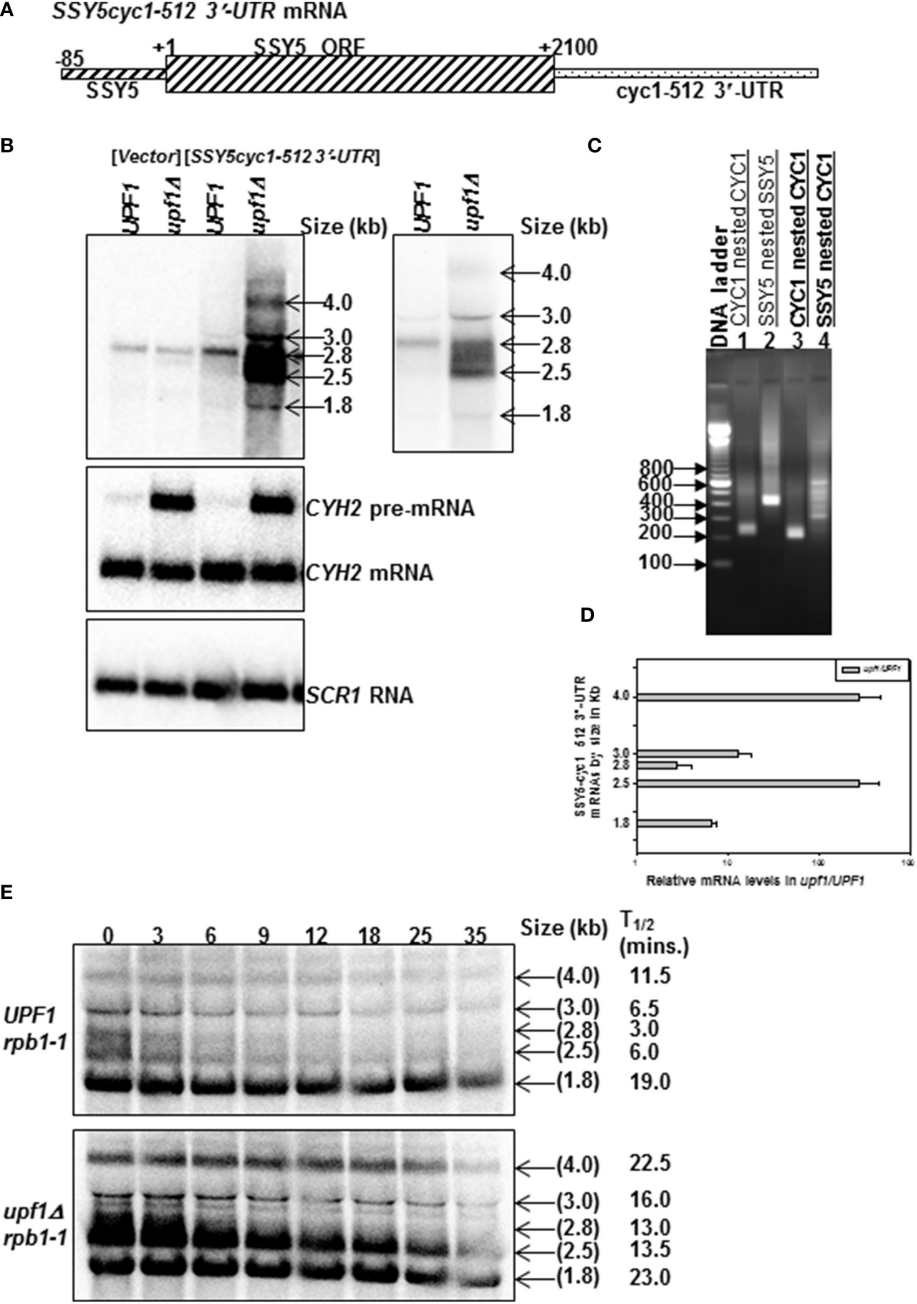
***SSY5* mRNA requires the 3′-UTR to escape from NMD-mediated degradation**. Replacement of the *SSY5* 3′-UTR with the *cyc1-512* 3′-UTR results in the production of multiple transcripts that a degraded by the NMD pathway. Schematic representation of the *SSY5cyc1-512* 3′-UTR fusion mRNAs **(A)**. Representative steady-state northerns of total RNA isolated from wild-type (*UPF1)* and nmd mutants (*upf1Δ)* are shown below the mRNA schematic **(B)**. A shorter exposure of the steady-state northern blot is shown to the right of the steady-state accumulation northern for clearer visualization of the bands. The two right lanes of the northern blot contain RNA from yeast strains expressing the *SSY5cyc1-512* 3′-UTR construct **(B)**. The sizes of the predominant bands are shown to the right of the northern blot. The relative *SSY5cyc1-512* 3′-UTR steady-state levels are represented graphically in log scale **(D)**. An agarose gel of 3′ RACE nested PCR products from an nmd mutant yeast strain lacking the *SSY5cyc1-512* 3′-UTR mRNAs (lanes 1–2) and expressing the *SSY5cyc1-512* 3′-UTR mRNAs (lane 3–4). Primers used for the 3′ RACE PCR reactions are listed above the lane numbers **(C)**. Half-life measurements of the *SSY5cyc1-512* 3′-UTR mRNAs are shown in **(E)** and are an average of at least two independent experiments. The northern blots were probed with DNA from the *SSY5 5*'*-UTR-ORF*, *CYH2* and *SCR1*.

We next tested whether the *SSY5* 5′-UTR and ORF are sufficient to protect the mRNA from degradation by the NMD pathway by replacing the *SSY5* 3′-UTR with the *cyc1-512* 3′-UTR. *cyc1-512* has a 38 nt deletion that results in defective 3′ end processing leading to the production of multiple transcripts with aberrant extended 3′-UTRs (Zaret and Sherman, 1982). These aberrantly extended cyc1 transcripts are degraded by the NMD pathway (Muhlrad and Parker, 1999). 3′-RACE was used to show that the 3′-UTR of the *SSY5* mRNA was specifically replaced with the *cyc1-512* 3′-UTR (Figure 3C and Supplementary Figure S2, compare lanes 5 and 11).

*SSY5* is an essential gene. Consequently the *SSY5cyc1-512* 3′-UTR transcripts were expressed from a high copy plasmid in wild-type and *nmd* mutant strains with *SSY5*. Multiple *SSY5cyc1-512* 3′-UTR transcripts were observed on northerns (Figures 3B,E). The *SSY5* transcript is ~2.8 kb (Figure 3B, vector control; Kebaara and Atkin, 2009). The endogenous *SSY5* mRNA accumulated 2.8-fold and ~133-fold lower than the most abundant *SSY5cyc1-512* 3′-UTR transcript in *nmd* mutant strains (Figures 3B,D). The *SSY5cyc1-512* 3′-UTR transcripts are at different positions on the northern blots because they have 3′-UTRs that differ in length correlating with the heterogeneous bands observed by 3′-RACE (Figures 3B,C, lane 4). The major transcripts ranged in size from ~1.8 to 4 kb (Figure 3B). The 1.8 kb transcript could be a non-stop transcript or a decay intermediate because the *SSY5* ORF is 2100 nt. Notably, while the levels of these transcripts varied within a strain, they consistently accumulated to higher levels in *nmd* mutant strains relative to wild-type strains. The relative fold changes of the *SSY5cyc1-512* 3′-UTR transcripts are represented graphically in Figure 3D.

All but the ~1.8 kb major *SSY5cyc1-512* 3′-UTR fusion mRNAs had shorter half lives in the wild-type cells than the *nmd* mutant (Figure 3E). The shortest transcript detected of ~1.8 kb decayed at comparable rates in wild-type and *nmd* mutants. This transcript is unlikely to contain the entire *SSY5* ORF of 2100 nt. The band that corresponds in size to the endogenous *SSY5* transcript also showed sensitivity to the NMD pathway (Figure 3E). The half-life of the *SSY5* mRNA in the *nmd* mutant strain was 3.0 ± 1.41 (*n* = 2) min vs. 13.0 ± 2.83 (*n* = 2) min in wild-type stains (Figure 3E). These half-life measurements are comparable to those observed for the longer ~2.8 kb *SSY5* transcript (Figure 1B). Thus, replacement of the *SSY5* 3′-UTR with a 3′-UTR known to target mRNAs to the NMD pathway results in the production of multiple mRNAs that were sensitive to NMD. These results demonstrate that the *SSY5* 5′-UTR and ORF are insufficient to stabilize the mRNA in the presence of the *cyc1-512 3′-UTR* and suggest that additional sequences within the *SSY5* 3′-UTR are required to escape degradation by the NMD pathway.

### The SSY5 mRNA requires the 5′-UTR, ORF and the 3′-UTR to escape the NMD pathway

**Figure 4:**
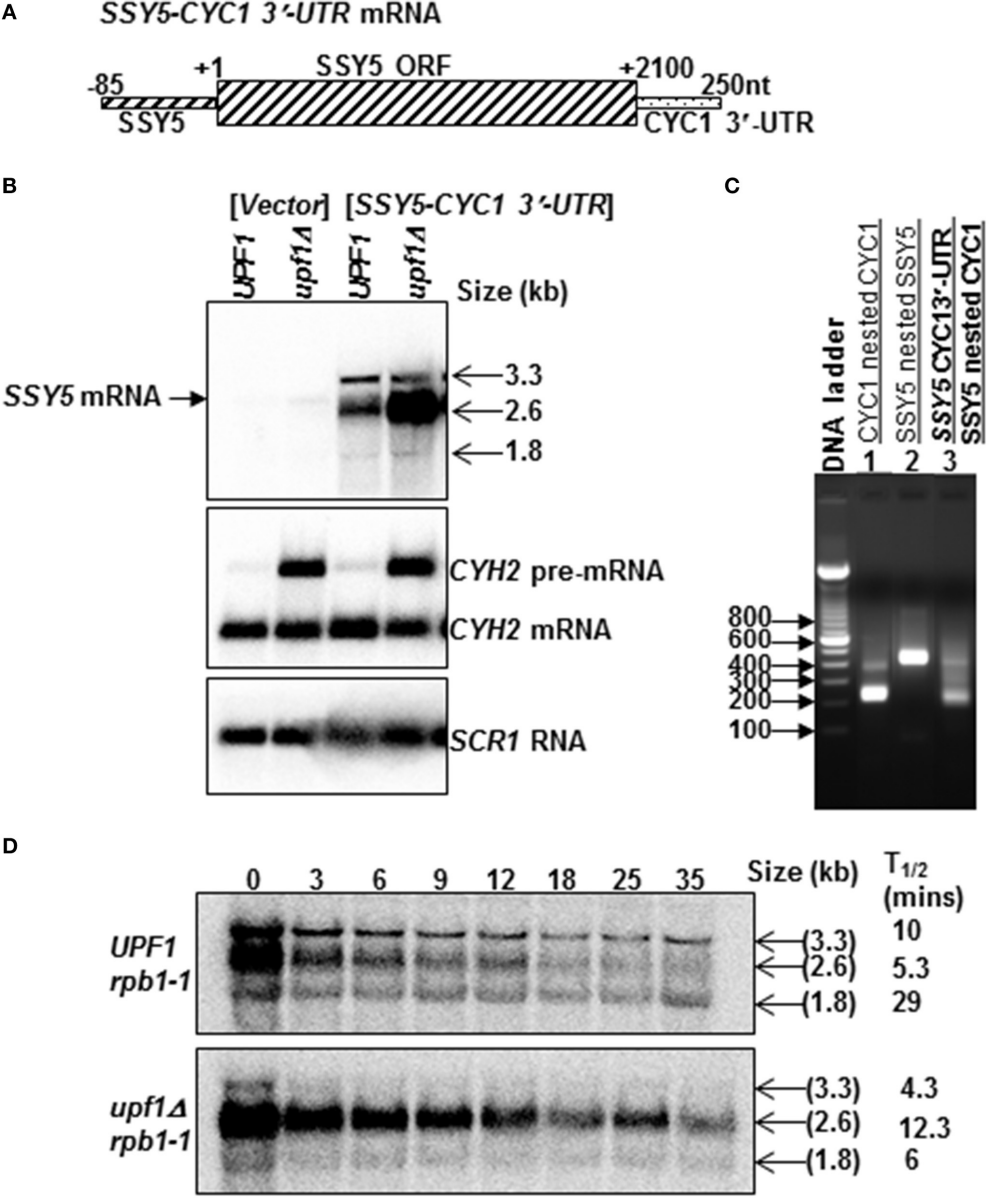
**Some mRNAs expressed from yeast strains transformed with the *SSY5-CYC1* 3′-UTR mRNA are sensitive to the NMD pathway**. Replacement of the *SSY5* 3′-UTR with the *CYC1* 3′-UTR resulted in the production of three transcripts that are differentially regulated by the NMD pathway. Schematic diagram of the *SSY5-CYC1* 3′-UTR fusion mRNA **(A)**. Representative steady-state northern blot of total RNA isolated from wild-type (*UPF1)* and nmd mutants (*upf1Δ)* are shown below the mRNA schematic. The first 2 lanes of the northern blot are from yeast strains lacking the *SSY5-CYC1* 3′-UTR mRNA **(B)**. The sizes of the *SSY5-CYC1* 3′-UTR transcripts are shown to the right of the northern blot **(B)**. An agarose gel of 3′ RACE nested PCR products from a wild-type *(UPF1)* yeast strain lacking the *SSY5-CYC1* 3′-UTR mRNA (lanes 1–2) and expressing the *SSY5-CYC1* 3′-UTR mRNA (lane 3). Primers used for the 3′ RACE PCR reactions are listed above the lane numbers **(C)**. Half-life measurements of the *SSY5-CYC1* 3′-UTR mRNAs are shown in **(D)** and are an average of three independent experiments. The northern blots were probed with DNA from the *SSY5 5*'*-UTR-ORF*, *CYH2*, and *SCR1*.

The observation that replacement of the *SSY5* 3′-UTR with a 3′-UTR known to target mRNA to the NMD pathway results in the production of multiple transcripts the majority of which are sensitive to NMD, led us to generate an additional fusion mRNA as a control. This control mRNA was utilized to determine whether removal of the *SSY5* 3′-UTR from the mRNA would result in stabilization of the mRNA. The *SSY5-CYC1* 3′-UTR mRNA was synthesized using cloning free PCR to precisely replace the *SSY5* 3′-UTR with the *CYC1* 3′-UTR (Figure 4A) and verified by 3′-RACE (Figure 4C, compare lanes 1 and 3). The *SSY5-CYC1* 3′-UTR transcripts were expressed from a high copy plasmid in wild-type and *nmd* mutant strains with *SSY5*. Three *SSY5-CYC1* 3′-UTR-specific bands, a major one of ~2.6 kb and two minor ones of ~1.8 kb and ~3.3 kb were detected on northern blots (Figure 4B). These bands were not expressed in the vector control and are much more abundant than the endogenous *SSY5* transcript (Figure 4B compare lanes 1 and 2 to 3 and 4).

We expected the *SSY5-CYC1* 3′-UTR mRNA to be equally stable in wild-type and *nmd* mutant cells, but this was not the case. The predominant ~2.6 kb fusion mRNA accumulated 7.59 ± 3.7 (*n* = 3) fold more in the *nmd* mutants than wild-type (*UPF1)* strains. The levels of the less abundant 3.3 kb and 1.8 kb transcripts were not significantly different in *nmd* mutant or wild-type strains [0.57 ± 0.09 (*n* = 3) and 1.5 ± 0.1 (*n* = 2), respectively]. The ~1.8 kb transcript was shorter than the expected size of the *SSY5-CYC1* 3′-UTR fusion mRNA, although it was unique to yeast strains expressing the *SSY5-CYC1 3′-UTR* fusion construct (Figure 4B).

The half-lives of all three *SSY5-CYC1* 3′-UTR mRNAs were determined (Figure 4D). The predominant ~2.6 kb transcript had half-lives of 5.3 ± 1.53 (*n* = 3) min in wild-type strains and 12.3 ± 0.57 (*n* = 3) min in *nmd* mutants and was thus degraded faster in wild-type cells than *nmd* mutants. The ~3.3 kb and shortest ~1.8 kb transcripts were not stabilized in *nmd* mutants. The half-lives of the 3.3 kb transcript were 10 ± 2.65 (*n* = 3) in wild-type strains and 4.3 ± 1.53 (*n* = 3) min in *nmd* mutants and the ~1.8 kb transcript had half-lives of 29 ± 5.29 (*n* = 3) min in wild-type strains and 6 ± 2 (*n* = 3) min in *nmd* mutants. Thus, both of these mRNAs had shorter half-lives in the *nmd* mutant than wild-type cells. This is a novel phenotype. These results show that the yeast strains expressing the *SSY5-CYC1* 3′-UTR fusion construct produce multiple transcripts. These transcripts are differentially affected by the NMD pathway; the 2.6 kb transcript was degraded by the NMD pathway, while the ~3.3 and 1.8 kb targets were stabilized by inactivation of the NMD pathway. As far as we know this is the first example of transcripts that are destabilized by inactivation of the NMD pathway.

Degradation of the 2.6 kb *SSY5-CYC1 3′-UTR* transcript by the NMD pathway suggests that the *SSY5* mRNA may contain alternative NMD targeting features besides the atypically long 3′-UTR, since in the absence of the long 3′-UTR from the *SSY5* transcripts generates some transcripts that are still sensitive to the NMD pathway. Examination of the *SSY5* mRNA showed that the start codon is in a suboptimal context and is followed by an out-of-frame AUG at +71, which is in an optimal context (Welch and Jacobson, 1999). This feature could potentially target the *SSY5* mRNA to NMD through leaky scanning. Additionally, the *SSY5* mRNA contains ribosomal frameshifting signals and has a translated uORF (Ingolia et al., 2009; Belew et al., 2010). These three features have been shown to target natural mRNAs to the NMD pathway in *S. cerevisiae* (Welch and Jacobson, 1999; Guan et al., 2006; Belew et al., 2010). Since these features are still present in the *SSY5-CYC1 3′-UTR* mRNA, one or more of these features could be targeting the *SSY5-CYC1 3′-UTR* mRNA for decay by the NMD pathway.

## Discussion

Here, we show that there are two *SSY5* transcripts. One that is expressed constitutively and a second that was only observed under specific conditions and yeast genetic backround. The constitutively expressed *SSY5* mRNAs has an atypically long 3′-UTR of ~475 nts and can escape degradation by NMD in some conditions (Figure 1A). The *SSY5* mRNA produced in *rpb1-1* genetic background is shorter than the constitutively expressed *SSY5* mRNA and is immune to the NMD pathway. *SSY5* mRNAs contains multiple NMD targeting signals including a translated uORF, leaky scanning, programmed ribosome frameshift sites and a long 3′-UTR (Welch and Jacobson, 1999; Guan et al., 2006; Kebaara and Atkin, 2009; Belew et al., 2010). We showed that the ~475 nt 3′-UTR of the constitutive *SSY5* mRNA is sufficient to target an mRNA that is normally NMD-insensitive for decay by the pathway (Figure 2). And finally, we show that protection of the *SSY5* mRNA from decay by the NMD pathway requires sequences in the 5′-UTR-ORF and the 3′-UTR because *SSY5 cyc1 512* 3′-UTR and *SSY5-CYC1* 3′-UTR fusion RNAs were both degraded by the NMD pathway (Figures 3, 4). Collectively, we show that the *SSY5* mRNA contains multiple NMD targeting features; however this mRNA is immune to NMD-mediated degradation in some conditions and this immunity is due to the presence of elements in the 5′-UTR and/or ORF and the 3′-UTR.

Ssy5p is a component of the SPS (Ssy1-Ptr3-Ssy5) extracellular amino acid sensing system. It is a protease that cleaves the cytosolic precursor forms of the transcription factors Stp1p and Stp2p to activate transcription of amino acid permease genes. The *SSY5* mRNA is protected from NMD in yeast cells that are grown at normal growth temperature (30°C) and in rich medium (Kebaara and Atkin, 2009; Figure 1). We detected an additional transcript on Northern blots probed with a *SSY5* probe in *rpb1-1* yeast strains subjected to heat stress (Figures 1B,C). Surprisingly in these conditions the constitutively expressed *SSY5* transcript was degraded by the NMD pathway. In addition, manipulation of the *SSY5* mRNA coding and 3′ regulatory sequences leads to the production of multiple transcripts (Figures 3, 4). Some of these transcripts were regulated in an NMD-dependent manner (Figures 1, 4). Sensitivity of the *SSY5* mRNA to the NMD pathway is influenced by both the mRNA secondary structure and trans-acting factors that bind to the mRNA. Future studies on the *SSY5* messenger ribonucleoprotein (mRNP) composition will elucidate the role trans acting factor(s) function in the evasion of this mRNA from NMD. We hypothesize that the *SSY5* mRNP composition in different conditions is important in determining the fate of the *SSY5* mRNA, specifically whether the *SSY5* mRNAs will be sensitive to the NMD pathway.

A Previous study identified direct NMD targets in *S. cerevisiae* based on their association with Upf1p, a core component of the NMD pathway. This study found that the *SSY5* mRNAs associates with Upf1p (Johansson et al., 2007). Whether Upf1p binds to the *SSY5* mRNA before or after the decision to degrade the mRNAs by NMD is unclear. In mammalian cells it has been proposed that Upf1p binds to mRNAs before the decision on whether an mRNA will be degraded by the pathway or escape degradation by the pathway (Zund et al., 2013). If this scenario applies to the *SSY5* mRNA, then the decision on whether the *SSY5* mRNAs are degraded by NMD could occur after Upf1p binding.

Replacement of the *SSY5* 3′-UTR with the *cyc1-512* 3′-UTR resulted in the production of multiple transcripts the majority of which were regulated in an NMD dependent manner (Figure 3). If the *SSY5* mRNA contains a universal NMD stabilizer element in the 5′-UTR and/or ORF, we would expect that the transcripts generated from the *SSY5cyc1-512* 3′-UTR fusion mRNA would be insensitive to the NMD pathway, if proper 3′-end processing is not required to stabilize the transcripts. Regulation of these transcripts by the NMD pathway suggests that the *SSY5* mRNA stabilizer element requires sequences in both the 5′-UTR-ORF region and the 3′-UTR since neither regions was sufficient to stabilize the fusion RNAs, or that the functionality of the stabilizer element is context specific. Specifically, the stabilizer element stabilizes the *SSY5* mRNA in the presence of the *SSY5* 3′-UTR but not in the presence of the *cyc1-512* 3′-UTR.

Replacement of the *SSY5* 3′-UTR with the *CYC1* 3′-UTR resulted in the production of three transcripts. Two of the *SSY5-CYC1* 3′-UTR transcripts were unexpectedly degraded more rapidly in *nmd* mutants relative to wild-type yeast strains. NMD targets are normally degraded faster in wild-type yeast strains and stabilized in *nmd* mutants. The only transcript that was sensitive to the NMD pathway was the ~2.6 kb transcript, which was expressed at higher levels than the other two mRNAs and migrated slightly faster than the endogenous *SSY5* mRNA. These data show that fusion mRNAs produced from the same fusion construct can be differentially regulated by the NMD pathway, where some transcripts are sensitive to the pathway and others are immune to the pathway (Figure 4B). The explanation for the differential sensitivity of the *SSY5-CYC1* 3′-UTR transcripts to the NMD pathway is unclear but there are some possible explanations. First, the loss of part of the stabilizer element present in the *SSY5* 3′-UTR and also the presence of three additional NMD targeting features within the NMD sensitive transcript may result in differential regulation. Alternatively, the sensitivity of ~2.6 kb *SSY5-CYC1* 3′-UTR to the NMD pathway could be due to the titration of trans-acting factors required for decay of these mRNAs in wild-type cells. Interestingly, a recent study by Geisberg et al., showed that mRNAs isoforms whose 3′-ends differ by as few as 3 nucleotides have relatively different half-lives (Geisberg et al., 2014).

The role the *SSY5* ORF plays in the immunity of the mRNA to the NMD pathway was not specifically examined here. However, the *SSY5* ORF may contribute to the escape of the mRNA from the pathway due to a couple of observations. First, in our previous studies examining endogenous mRNAs with atypically long 3′-UTRs that are regulated by the NMD pathway, the *SSY5* mRNA was the only mRNA out of 11 with an atypically long 3′-UTR that was immune to the NMD pathway (Kebaara and Atkin, 2009). Notably, the *SSY5* mRNA also had the longest ORF, suggesting that the ORF length may play a role in the escape of the mRNA from the NMD. This is consistent with a recent study in *S. cerevisiae* that reported that mRNAs subject to NMD due to the presence of a long 3′-UTR had short ORFs and that increasing the ORF length of these mRNAs had a stabilizing effect (Decourty et al., 2014). The *SSY5* ORF length however does not fully account for all our observations because the *SSY5cyc512* 3′-UTR and *SSY5-CYC1* 3′-UTRs fusions contained the full length *SSY5* ORF, and yet the predominant transcripts encoded by these fusions were degraded by the NMD pathway. This indicates that there are additional requirement for protection.

In *S. cerevisiae two* additional mRNAs with NMD targeting features have been reported to escape degradation by the pathway, *GCN4* and *YAP1* mRNAs (Ruiz-Echevarria and Peltz, 2000). These mRNA have uORFs. mRNAs containing uORFs in the 5′-UTR or overlapping the ORF are normally degraded by the NMD pathway (Ruiz-Echevarria and Peltz, 2000; Gaba et al., 2005; Guan et al., 2006; Yun et al., 2012). The immunity of *GCN4* and *YAP1* mRNA to NMD is reported to be dependent on the presence of Pub1p (Ruiz-Echevarria and Peltz, 2000). We hypothesize that specific trans-acting factors may regulate the degradation of *SSY5* mRNA by NMD. In mammalian cells, a number of mRNAs with atypically long 3′-UTRs have been reported to escape NMD-mediated degradation (Singh et al., 2008). Similarly, these mRNAs may have also evolved mechanisms to avoid degradation by NMD. Collectively, the results presented here demonstrate that naturally occurring transcripts with multiple NMD targeting features can escape NMD-mediated degradation in some conditions. These mRNAs escape degradation because they have evolved mechanisms to prevent degradation by NMD. These NMD evasion mechanisms maybe particular for specific transcripts and may also be context specific.

## Author contributions

Jesseeca Obenoskey and Dakota Lane contributed to the acquisition and analysis of data. Audrey Atkin contributed to the conception of the research and revising the manuscript. Bessie Kebaara contributed to conception, acquisition and analysis of data, drafting and revising the manuscript and final approval of the version to be published.

## Funding

This work was supported by grants from the National Science Foundation (MCB-0444333 to Audrey L. Atkin, RIG-0642154 to Bessie W. Kebaara) and start-up funds from Baylor University. Any opinions, findings, conclusions, or recommendations expressed in this report are ours, and do not necessarily reflect the views of the National Science Foundation.

### Conflict of interest statement

The authors declare that the research was conducted in the absence of any commercial or financial relationships that could be construed as a potential conflict of interest.
