# Supplementary material for: Immunity of the Saccharomyces cerevisiae SSY5 mRNA to nonsense-mediated mRNA decay
Source: Front Mol Biosci. 2014 Dec 8;1:25. doi: 10.3389/fmolb.2014.00025 (PMC4428434; doi:10.3389/fmolb.2014.00025)
Supplement: Supplementary file 1 [file DataSheet1.DOCX]

**SUPPLEMENTARY INFORMATION**

**Supplementary Figure 1.** Replacement of the *CYC1* 3′-UTR with the *SSY5* 3′-UTR was confirmed by 3′RACE. Supplementary Figure 1 is a 1.5% agarose gel that contains 20µl of the primary and nested PCR products listed above the gel. The primers used for the primary and nested PCR reactions are listed above the lane numbers. The DNA products from the nested PCR reactions are underlined. The primers used for each PCR reaction are shown on the schematic diagrams and correspond to the lane numbers above the gel. Lane **1** shows the primary *CYC1* 3′-RACE PCR products using cDNA from a wild-type yeast strain. Lane **2** and **3** are the nested *CYC1* PCR products, lane **2** is nested with *CYC1* and shows the ~250 nt band that corresponds to the *CYC1* 3′-UTR. Lane **3** is the primary *CYC1* 3′ RACE PCR products nested with *SSY5*. The white arrow points to the lack of the *SSY5* band when the primary *CYC1* 3′ RACE PCR products are nested with *SSY5*, in the absence of the *CYC1-SSY5 3′-*UTR fusion construct. Lane **4** shows the primary *SSY5* 3′-RACE PCR products generated using cDNA from a wild-type yeast strain. Lane **5** is the primary *SSY5* 3′-RACE PCR product nested with SSY5. Lanes **4** and **5** show a band of ~475 nt which corresponds to the *SSY5* 3′-UTR. Lanes **6-8** show the 3′-RACE PCR products from cDNA of a wild-type yeast strain expressing the *CYC1-SSY53′-UTR* fusion construct and are shown in bold type. Lane **6** shows the 3′-RACE PCR product using the *CYC1* primer, three prominent bands are observed. Lane **7** shows the *CYC1* primary 3′-RACE PCR product nested with *CYC1*. In lane **7**, a band of ~250 nt is produced from the endogenous CYC1 mRNA. Lane **8** shows the *CYC1-SSY53′-UTR* primary 3′-RACE PCR product nested with *SSY5*. In lane **8**, a band of ~475 nt representing the *SSY5* 3′-UTR is produced from the *CYC1-SSY53′-UTR* fusion mRNA. This band is specific to the *CYC1-SSY53′-UTR* fusion mRNA because it is absent from lane **3** as shown by the white arrow**.** The nested PCR products generated in lane **3** are from a wild-type strain lacking the *CYC1-SSY53′-UTR* fusion mRNA.

**Supplementary Figure 2.** Replacement of the *SSY5* 3'-UTR with the *cyc1-512* 3'-UTR

Supplementary Fig 2 shows a 1.5% agarose gel of 20µl of primary and nested 3′-RACE PCR products. The primers used for the primary and nested PCR reactions are listed above the lane numbers. The nested PCR products are underlined, while the primary and nested PCR expressed from the *cyc1-512* 3'-UTR fusion mRNAs are shown in bold. The primers used for each PCR reaction are shown on the schematic diagrams and correspond to the lane numbers above the gel Lanes **1-3** show the *CYC1* mRNA 3′ RACE PCR products from cDNA of an nmd mutant yeast strain. Lane **1** contains the primary *CYC1* 3′ RACE PCR products, while lanes **2** and **3** show the *CYC1* primary PCR products nested with *CYC1* or *SSY5* primers respectively. Lane **2** shows the ~250 nt *CYC1* 3′-UTR and Lane **3** shows the absence of the *CYC1* 3′-UTR because the primary *CYC1* 3′-RACE PCR products are nested with SSY5 primer. Lanes **4-6** show the *SSY5* mRNA 3′ RACE PCR products. Lane **4** shows the primary *SSY5* 3′ RACE PCR products, while lanes **5** and **6** are of the *SSY5* primary PCR products nested with *CYC1* or *SSY5* primers respectively. In lane **5** the white bracketed area shows the absence of ~300-650 nt 3' RACE PCR products when the primary *SSY5* 3′ RACE PCR products are nested with *CYC1* in the absence of the *cyc1-512* 3'-UTR fusion mRNA. Lanes **7-12** contain 3′-RACE PCR products from nmd mutant yeast strains expressing the *cyc1-512* 3'-UTR fusion construct. Lanes **1-6** lack the *cyc1-512* 3'-UTR fusion mRNA. The primary PCR products in Lane **7** were generated using the *CYC1* 3'-RACE primer. The primary PCR products were then nested with the *CYC1* nested primer (lane **8**) or the *SSY5* nested primer (lane **9**). Lane **8** shows a ~250 nt PCR product that corresponds to the endogenous *CYC1* mRNA 3'-UTR. Lane **9** shows multiple nonspecific bands. The primary PCR products in lane **10** were generated using the *SSY5* 3'-RACE primer. The primary PCR products were then nested with the *CYC1* nested primer (lane **11**) or the *SSY5* nested primer (lane **12**). In lane **11** the bracketed area shows bands of ~300-650, that are missing from lane **5**. Lane **5** contains PCR products from the identical PCR reaction in the absence of the *SSY5-cyc1-512* 3'-UTR mRNA. Lane **12** shows a band, ~475 nt, which corresponds to the endogenous *SSY5* 3'-UTR.

**Supplementary Fig 3.** Replacement of the SSY5 3′-UTR with the CYC1 3′-UTR. Supplementary Fig 3 shows a 1.5% agarose gel of 20µl of primary and nested 3′-RACE PCR products. The primers used for the primary and nested PCR reactions are listed above the lane numbers. The primers used for each PCR reaction are also shown on the schematic diagram and correspond to the lane numbers above the gel The nested PCR products are underlined, while the primary and nested PCR generated from the *SSY5-CYC1* 3′-UTR fusion mRNA are shown in bold. Lanes **1-3** show the *CYC1* mRNA 3′ RACE PCR products from cDNA of a wild-type yeast strain. Lane **1** contains the primary *CYC1* 3′ RACE PCR products, while lanes **2** and **3** are the *CYC1* primary PCR products nested with *CYC1* or *SSY5* primers respectively. Lane **2** shows the ~250 nt *CYC1* 3′-UTR and Lane **3** shows the lack of the *CYC1* 3′-UTR because the primary *CYC1* 3′-RACE PCR products are nested with the *SSY5* primer. Lanes **4-6** show the *SSY5* mRNA 3′ RACE PCR products. Lane **4** is the primary *SSY5* 3′ RACE PCR products, while lanes **5** and **6** are the *SSY5* primary PCR products nested with *CYC1* or *SSY5* primers respectively. In lane **5** the white arrow is pointing to the absence of ~250nt PCR product when the primary *SSY5* 3′ RACE PCR products are nested with *CYC1* in the absence of the *SSY5-CYC1* 3′-UTR fusion mRNA. Lanes **7-12** contain 3′-RACE PCR products using cDNA from a yeast strain expressing the *SSY5-CYC1* 3′-UTR fusion mRNA, Lanes **1-6** lack the *SSY5-CYC1* 3′-UTR fusion mRNA. The primary PCR products in Lane **7** were generated using the *CYC1* 3 '-RACE primer. The primary PCR products were then nested with the *CYC1* nested primer (lane **8**) or the *SSY5* nested primer (lane **9**). Lane **8** shows a ~250 nt PCR product that corresponds to the endogenous *CYC1* mRNA 3′-UTR. Lane **9** shows the ~475 nt *SSY5* PCR product that corresponds to the endogenous *SSY5* mRNA 3'-UTR. The primary PCR products in lane **10** were generated using the *SSY5* 3′-RACE primer. The primary PCR products were then nested with the *CYC1* nested primer (lane **11**) or the *SSY5* nested primer (lane **12**). Lane **11** shows two major bands, one of ~475 nt, which corresponds to the endogenous *SSY5* mRNA. The second band of ~250 nt corresponds to the *SSY5-CYC1* 3′-UTR fusion mRNA because it is absent from the 3′RACE PCR products in lane **5** which does not express the *SSY5-CYC1* 3′-UTR fusion mRNA. Lane **12** shows a band, ~475 nt, which corresponds to the endogenous *SSY5* 3'-UTR.
