# Supplementary figures and images for: Immunity of the Saccharomyces cerevisiae SSY5 mRNA to nonsense-mediated mRNA decay
Source: Front Mol Biosci. 2014 Dec 8;1:25. doi: 10.3389/fmolb.2014.00025 (PMC4428434; doi:10.3389/fmolb.2014.00025)

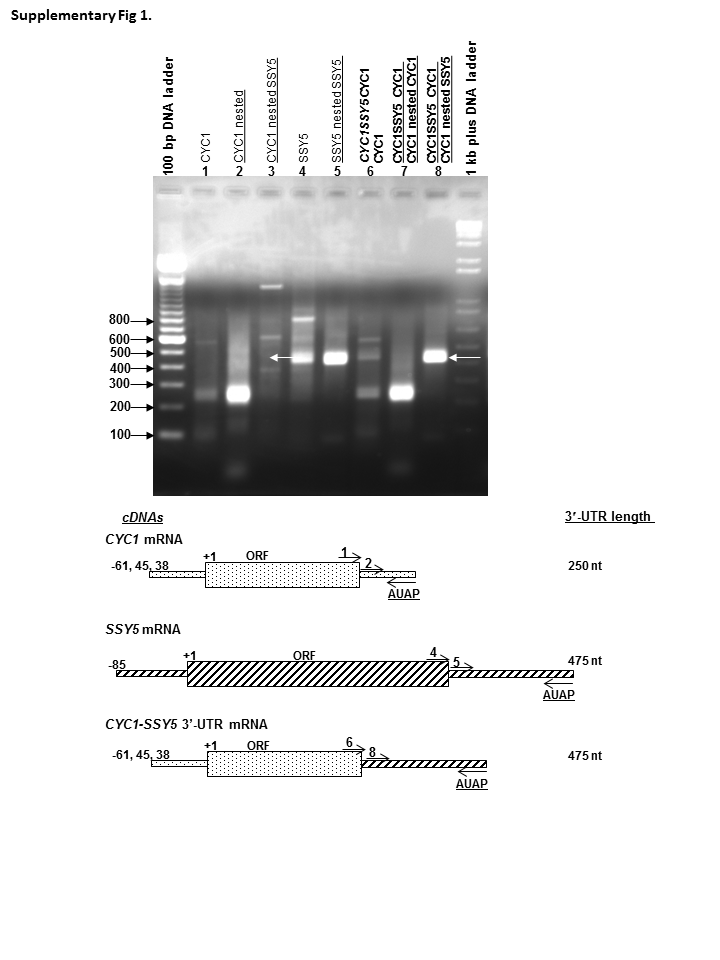

Supplement: Supplementary file 2 [file Image1.TIF]

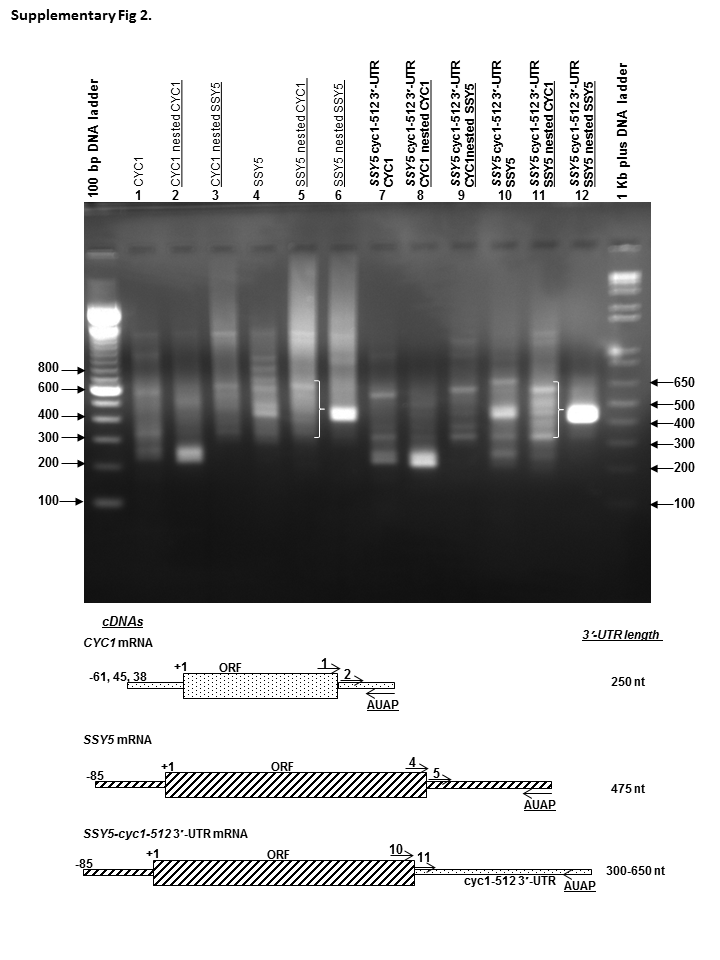

Supplement: Supplementary file 3 [file Image2.TIF]

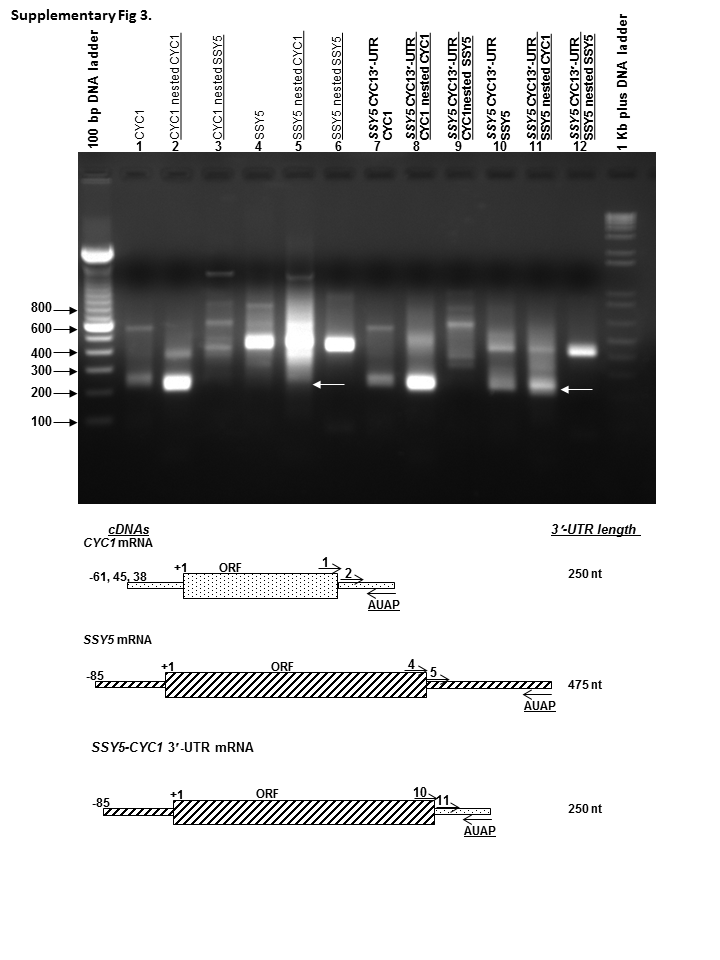

Supplement: Supplementary file 4 [file Image3.TIF]
